# A review of therapeutic impacts of saffron (*Crocus sativus* L.) and its constituents

**DOI:** 10.14814/phy2.15785

**Published:** 2023-08-03

**Authors:** Fatemeh Anaeigoudari, Akbar Anaeigoudari, Aliasghar Kheirkhah‐Vakilabad

**Affiliations:** ^1^ Student Research Committee, Afzalipour Faculty of Medicine Kerman University of Medical Sciences Kerman Iran; ^2^ Department of Physiology, School of Medicine Jiroft University of Medical Sciences Jiroft Iran; ^3^ Clinical Research Development Center of Imam Khomeini Hospital Jiroft University of Medical Sciences Jiroft Iran

**Keywords:** *Cricus sativus*, crocin, inflammation, oxidative stress, saffron

## Abstract

Application of herbal medicines in the treatment of diseases is in the center of attention of medical scientific societies. Saffron (*Cricus sativus L*.) is a medicinal plant belonging to the Iridaceae family with different therapeutic properties. The outcomes of human and animal experiments indicate that therapeutic impacts of saffron and its constituents, crocin, crocetin, and safranal, mainly are mediated via inhibiting the inflammatory reactions and scavenging free radicals. It has been suggested that saffron and crocin extracted from it also up‐regulate the expression of sirtuin 1 (SIRT1) and nuclear factor erythroid 2‐related factor 2 (Nrf2), down‐regulate nuclear factor kappa B (NF‐κB) signaling pathway and untimely improve the body organs dysfunction. Inhibition of inducible nitric oxide synthase and cyclooxygenase‐2 (COX2) also is attributed to crocin. The current review narrates the therapeutic effects of saffron and its constituents on various body systems through looking for the scientific databases including Web of Science, PubMed, Scopus, and Google Scholar from the beginning of 2010 until the end of 2022.

## Graphical abstract

The protective effects of saffron and its ingredients on various systems mainly are carried out by modulation of oxidative stress and suppression of inflammation reactions.

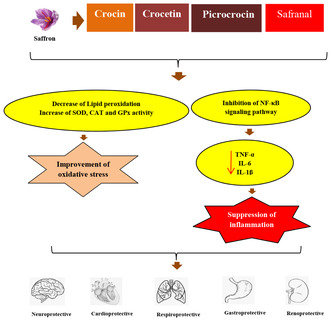

## 1 INTRODUCTION

**FIGURE 1:**
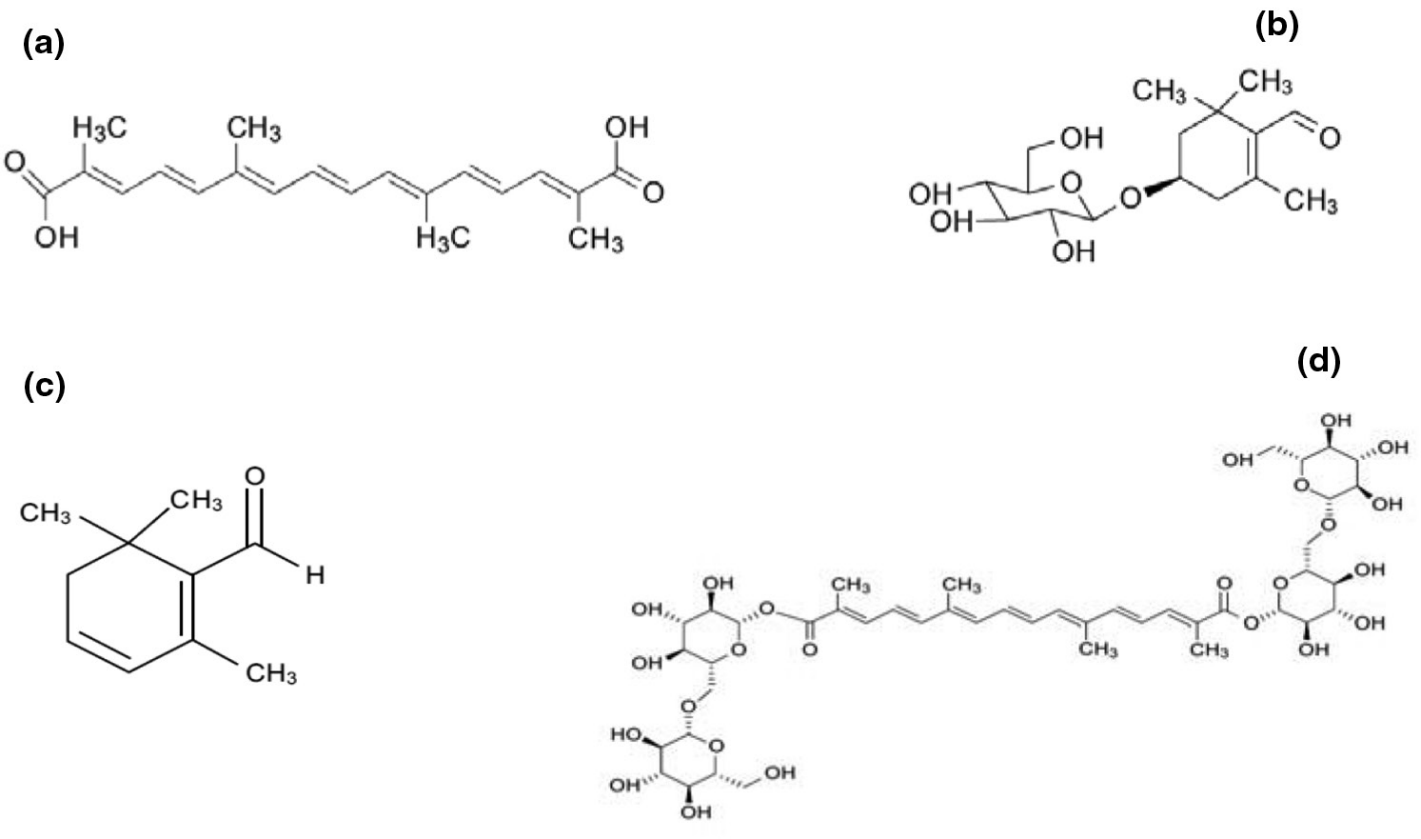
Molecular structure of (a) crocetin, (b) picrocrocin, (c) safranal, and (d) crocin.

Despite the widespread use of synthetic drugs for treatment of ailments, traditional medicine and application of phytochemicals also are in the center of attention of medical scientific societies (Khazdair, Anaeigoudari, Hashemzehi, et al., 2019). It is due to that traditional medicines are cheaper, more available, and have fewer side effects than modern drugs (Dalli et al., 2022). Saffron (*Cricus sativus L*.) is a medicinal plant belonging to the Iridaceae family which is cultivated in different areas of Asian, Africa, and Europe (Bukhari et al., 2018). It is a perennial plant with 30 cm in height, long, and delicate leaves and cup‐shaped purple flowers. This herbaceous plant tolerates the sunshine and temperature lower than 15°C and can grow in clayey and calcareous soils with a pH between 6 and 7 (El Midaoui et al., 2022). The pistils of saffron flowers possess three reddish‐orange stigmas with a pleasant aromatic smell. After picking the flowers by hand, the stigmas are separated, dried, and employed as saffron (Roshanravan & Ghaffari, 2022). Phytochemical analysis has been confirmed the presence of several volatile and nonvolatile compounds in the stigmas. Besides minerals, proteins, sugar, and vitamins such as B1 and B2, saffron possesses four main bioactive ingredients including crocin, crocetin, picrocrocin, and safranal (Al‐Snafi, 2016; Figure 1). Crocin and crocetin are carotenoid compounds causing yellow color of saffron. The flavor of saffron is due to picrocrocin whereas its specific odor is attributed to safranal (El Midaoui et al., 2022). Alongside use as a food spice, saffron has been shown to be beneficial in alleviation of various ailments. The results of animal studies confirm the antioxidant, anti‐inflammatory, anticancer, antidiabetic, and antihypertensive activities of saffron (Azami et al., 2021). Antioxidant and anti‐inflammatory properties of saffron mainly are related to crocin presented in the stigmas of saffron (Cerdá‐Bernad et al., 2022). It has been also recognized that crocetin and crocin also suppresses amyloid‐β aggregation and can be effective in attenuation of Alzheimer's disease (AD) symptoms (Ghahghaei et al., 2013; Tiribuzi et al., 2017). Safranal as a monoterpene aldehyde extracted from essential oil of the saffron also possesses several biological activities including antihyperglycemic (Samarghandian et al., 2013), anti‐inflammatory (Hazman & Bozkurt, 2015), antioxidant (Farahmand et al., 2013), anti‐seizure (Bo‐Qiang et al., 2018), and anxiolytic (Pitsikas, 2016) properties. The current review will narrate the therapeutic effects of saffron and its constituents on various body systems.

## 2 METHOD

The electronic databases employed for gathering the scientific reports included Web of Science, PubMed, Scopus, and Google Scholar. The human and animal reports were checked from the beginning of 2010 until the end of 2022 using keywords including “saffron” or “*crocus sativus*” or “crocin” or “crocetin” or “safranal” and “brain” or “nervous system” or “cardiovascular system” or “respiratory system” or “gastrointestinal tract” or “urinary system.”

## 3 EFFECTS ON NERVOUS SYSTEM

Biochemical balance of brain tissue is essential for normal function of the central nervous system (Birla et al., 2020). One of the factors causing brain biochemical disturbance is oxidative stress (Bilgiç et al., 2023). Abundant scientific findings are conforming that the oxidative stress and neuroinflammation are involved in the induction and progression of neurodegenerative diseases such as AD, Parkinson's disease, and neuropsychiatric disorders including anxiety and depression (Rehman et al., 2022). Medicinal plants extract and its ingredients have been understood to be as modulators of oxidative status and inflammation in the brain (Anaeigoudari, 2022b). Crocin is biochemical compound extracted from saffron extract with potent antioxidant and anti‐inflammatory properties (Abdulkareem Aljumaily et al., 2021). In an animal model of neurotoxicity induced by acrylamide, systemic administration of 12.5, 25, and 50 mg/kg of crocin could protect the rats' brain against oxidative stress by lowering the level of malondialdehyde (MDA) and enhancing the content of glutathione (GSH) (Mehri et al., 2015). Scientific reports indicated that peripheral injection of 20 mg/kg/day of crocin and crocin‐loaded niosomes also applied fruitful effects on brain oxidative damage resulting from paraquat in rats (Daneshvar et al., 2022). Safranal is a potent ingredient found in saffron petals with antioxidant, anti‐inflammatory (Alayunt et al., 2019), and anti‐anxiety (Pitsikas, 2016) activity. Researchers studied the intraperitoneal effect of 72.5, 145 mg/kg of safranal on brain injuries resulting from transient cerebral ischemia in rats. In this study, improving effect of safranal against cerebral ischemia is associated with increasing the antioxidant capacity and decreasing the lipid peroxidation in hippocampal tissue of rats (Sadeghnia et al., 2017). Sadeghnia et al. also reported that peripheral administration of 72.75, 145.5, and 291 mg/kg of safranal rescued the rat hippocampus from neurotoxicity caused by quinolinic acid (QA). They demonstrated that the level of MDA in groups treated by 145.5 and 291 mg/kg of safranal was lower and total thiol concentration was higher than group exposed by QA (Sadeghnia et al., 2013).

Traumatic brain injury (TBI) is an intracranial injury induced by an external force. Based on intensity, TBI is divided into mild traumatic brain injury (mTBI) and severe TBI (Khellaf et al., 2019). It has been demonstrated that after TBI, the generation of reactive oxygen species (ROS) enhances and immune cells activated start releasing inflammatory cytokines (Wang et al., 2020). In mouse model of mTBI, use of 50 mg/kg of saffron extract and 30 mg/kg of crocin could ameliorate behavioral and cognitive deficits through mitigating the inflammation and oxidative stress of brain tissue (Salem et al., 2022). The NOD‐like receptor protein 3 (NLRP3) inflammasome is considered to be as a key factor in triggering inflammatory responses (Wang & Hauenstein, 2020). Furthermore, sirtuin 1 (SIRT1) is an endogenous protective agent which regulates expression of inflammatory mediators including tumor necrosis factor alpha (TNF‐α) and interleukin (IL)‐1β (Ma et al., 2021). It has been recognized that inhibition of SIRT1 leads to oxidative stress and inflammation (Tu et al., 2021). Based on the results of study conducted by Shaheen et al. (2021) injection of 50 mg/kg of saffron extract attenuated brain injuries excited by mTBI in mice by suppressing the activity of NLRP3 and up‐regulation of SIRT1 expression.

Depression is a well‐known neurological disorder affecting the quality of life of many people in the world. Sadness, lack of motivation, insomnia, and anorexia have been listed as symptoms of depression (Anaeigoudari, 2022a). Previous studies have confirmed the relationship between change in level of many brain neurotransmitters and pathophysiology of depression. Serotonin (5‐hydroxytryptamine, 5‐HT) is brain neurotransmitter that plays outstanding role in the regulation of some physiological functions such as mood, learning and memory, pain, sleep, and appetite (Strasser et al., 2016). Findings are supporting that brain concentration of serotonin in depressed patients is lower with respect to healthy individuals (Molendijk et al., 2011). It has also been recognized that there is a relationship between the brain levels of neurotropic factors including brain‐derived neurotrophic factor (BDNF) and depression (Anaeigoudari, 2022a). In addition, there is a strong link between increased level of the inflammatory markers such as C‐reactive protein (CRP) and depression (Zhang et al., 2022). In a randomized clinical trial, the therapeutic effect of 1 mg of saffron for 1 month on depressive patients was evaluated. The results of the patient health questionnaires 9 (PHQ‐9) score showed a considerable therapeutic effects after 1 month of treatment by saffron. According to the biochemical analysis, improvement of depression symptoms was associated with reduced level of CRP, increased production of BDNF, and incremented access brain to serotonin (Ahmad et al., 2022). In an experimental model of cerebral ischemia, injection of 30, 100, 300 mg/kg of saffron extract improved anxiety‐like behaviors and cognitive deficits. Neuroprotective effect of saffron was mediated via alleviating astrogliosis and glial scar, reducing the level of IL‐6 and IL‐1β and elevating the concentration of IL‐10 (Zhong et al., 2020). MK‐801 as a strong noncompetitive N‐methyl‐[D]‐aspartate (NMDA) receptor antagonist disturbs behavioral functions and is used for inducing schizophrenia‐like behaviors in animals' models (Liang et al., 2022). Sun et al reported that 25 and 50 mg/kg of crocin modulated schizophrenia‐like symptoms in rats exposed by MK‐801. The ameliorative effects of crocin were accompanied with elevated expression of SIRT1 and BDN and alleviation of oxidative stress in hippocampus tissue (Sun et al., 2020). In a rat model of anxiety and depressive‐like behaviors caused by unpredictable chronic mild stress, 30 mg/kg of crocin also could ameliorate sickness behaviors by diminishing serum level of inflammatory cytokines such as TNF‐α and IL‐6, MDA, and corticosterone (Abbaszade‐Cheragheali et al., 2022). The effects of saffron and its constituents on nervous system were compacted in Table 1.

**TABLE 1:** The effects of saffron and its constituents on nervous system.

Treatment	Kind of study	Dose	Mechanism (s) effects	References
Crocin	Animal	12.5, 25, and 50 mg/kg	Reduction level of MDA and enhancement of the content of GSH	Mehri et al. (2015)
Crocin	Animal	20 mg/kg	Modulation of brain oxidative stress	Daneshvar et al. (2022)
Safranal	Animal	72.5, 145 mg/kg	Potentiation of antioxidant capacity and decrease the lipid peroxidation	Sadeghnia et al. (2017)
Safranal	Animal	145.5 and 291 mg/kg	Reduction of MDA level and increased total thiol concentration	Sadeghnia et al. (2013)
Saffron and crocin	Animal	50 and 30 mg/kg	Amelioration of behavioral and cognitive deficits through mitigating the inflammation and oxidative stress	Salem et al. (2022)
Saffron	Animal	50 mg/kg	Suppression of NLRP3 activity and upregulation of SIRT1 expression	Shaheen et al. (2021)
Saffron	Human	1 mg	Improvement of depression symptoms by reducing CRP level and upregulating of BDNF expression and increasing brain availability to serotonin	Ahmad et al. (2022)
Saffron	Animal	30, 100, 300 mg/kg	Improvement of anxiety‐like behaviors and cognitive deficits via alleviating astrogliosis and glial scar, reducing the level of IL‐6 and IL‐1β and elevating the concentration of IL‐10	Zhong et al. (2020)
Crocin	Animal	25 and 50 mg/kg	Modulation of schizophrenia‐like symptoms through elevating of SIRT1 and BDN expression and alleviating oxidative stress	Sun et al. (2020)
Crocin	Animal	30 mg/kg	Amelioration of sickness behaviors by diminishing serum level of inflammatory cytokines such as TNF‐α and IL‐6, MDA, and corticosterone	Abbaszade‐Cheragheali et al. (2022)

Abbreviations: BDNF, brain‐derived neurotrophic factor; CRP, C‐reactive protein; GSH, glutathione; IL, interleukin; MDA, malondialdehyde; NLRP3, NOD‐like receptor protein 3; SIRT1, sirtuin1; TNF‐α, tumor necrosis factor alpha.

## 4 EFFECTS ON CARDIOVASCULAR SYSTEM

Cardiovascular diseases (CVDs) have been considered to be as one of important causes of death in the word. One of the risk factors affecting CDVs is atherosclerosis (Tervaert, 2013). It has been documented that natural remedies can exert anti‐atherosclerotic effects (Zhang et al., 2020). In human study, use of 100 mg/day of saffron for 6 weeks ameliorated quality of life and appetite in atherosclerosis patients. In treated patients by saffron, the physical domain and social domain of quality of life enhanced in comparison with those of treated by placebo (Ahmadikhatir et al., 2022). In diabetic atherosclerotic mice, 30, 60, and 90 mg/kg/day of aqueous extract of saffron in a dose‐dependent manner applied anti‐atherosclerotic effects through stabilizing the atherosclerotic plaques and modulating the inflammatory reactions (Christodoulou et al., 2018).

Hypertension is also listed as a crucial risk factor that can disturb the normal function of cardiovascular system. Uncontrolled hypertension lowers the quality of life and shortens the life span of people (Colafella & Denton, 2018). In a human study on men aged 60–70 years with hypertension, 200 mg of saffron a long with resistance training decreased systolic blood pressure, diastolic blood pressure, and mean arterial pressure. This improving effect of saffron on blood pressure was accompanied with a significant increment in nitric oxide (NO) and adiponectin level and a noticeable reduction in endothelin‐1 concentration (Hooshmand‐Moghadam et al., 2021). In a randomized controlled clinical research achieved by Mojtahedi et al. (2022), also consumption of 200 mg of saffron in combination with resistance training ameliorated inflammation and reduced risk factors causing CVDs in old hypertensive men. The renin angiotensin system (RAS) is a hormonal system that is involved in regulation of vascular resistance and blood pressure (Durante et al., 2012). Malfunction of this system and high blood level of its main effector, angiotensin II (Ang II), develop hypertension and damage cardiovascular system (Navar et al., 2011). In animal study, intravenous injection of 10, 20, and 40 mg/kg of hydroalcoholic extract of saffron restored Ang II‐induced hypertension in rats. In this study, systolic blood pressure, mean arterial blood pressure, and heart rate in rats treated by extract were lower than those of Ang II group (Plangar et al., 2019). In a similar research, intravenous administration of 50, 100, and 200 mg/kg of crocin also could normalize high blood pressure caused by Ang II administration in rats by reducing systolic blood pressure, mean arterial blood pressure, and heart rate (Shafei, Faramarzi, et al., 2017).

A large number of studies demonstrate that oxidative stress is one of the most important reasons misadjusting the normal condition of cardiovascular system (Choi et al., 2018). Disruption of cardiac contractility resulting from the dysfunction of proteins responsible for excitation–contraction coupling has been attributed to overproduction of free radicals and reduction of antioxidants in myocardial tissue (Cadenas, 2018). Alongside oxidative stress, inflammation also is a consequential cause for emersion and development of CVDs (Garcia et al., 2019). In patients with coronary artery diseases, the ameliorating effect of 30 mg/day of crocin and 30 mg/day of aqueous extract of saffron for 8 weeks by assessing oxidants, antioxidants, and inflammatory parameters was done. The results indicated that crocin increased the expression of SITR1 and 5′ adenosine monophosphate‐activated protein kinase (AMPK), decreased the production of Lectin‐like oxidized low‐density lipoprotein (LDL) receptor‐1 (LOX1), nuclear factor kappa B (NF‐κB), and oxidized LDL (ox‐LDL). In addition, both crocin and saffron extract decremented monocyte chemoattractant protein‐1 (MCP‐1) level (Abedimanesh et al., 2020). It has been understood that the use of 200, 400, and 800 mg/kg of saffron extract relieved myocardial damages resulted from isoproterenol in rats through balancing oxidative status and maintaining hemodynamic functions of cardiovascular system (Sachdeva et al., 2012). In rats exposed by isoproterenol, administration of 20 mg/kg/day of crocin also improved hemodynamic of cardiovascular system and reinforced antioxidant defense (Goyal et al., 2010). It has also been indicated that 100 mg/kg/day of crocin attenuated periodontitis‐induced cardiac injury by the mitigation of MDA and enhancement of GSH, superoxide dismutase (SOD), catalase (CAT) in rats (Kocaman et al., 2021). Wang et al. (2018) claimed that intraperitoneal injection of 20, 40, and 60 mg/kg of crocin alleviated myocardial infarction‐mediated injuries in rats via lessening the concentration of MDA and NO and amplifying the SOD activity. The data extracted from the study carried out by Razavi et al. also elucidated the positive impacts of 25 and 50 mg/kg of crocin against subchronic diazinon‐triggered cardiotoxicity in rats. This cardioprotective effect of crocin was linked to its antioxidant and antiapoptotic properties (Razavi et al., 2013). In addition, treatment with 100 mg/kg of saffron exerted a positive therapeutic effect against lethal ventricular arrhythmias caused by heart reperfusion in the rats. The findings of this study corroborate that the anti‐arrhythmic effect of saffron likely is mediated through prolonging the effective refractory period of cardiac cells and preventing the oxidative stress (Joukar et al., 2013). It has been recognized that the production of ROS and accumulation of oxidant agents including MDA take place during myocardial ischemia–reperfusion injury (MIRI; Tian et al., 2019). In rat model of MIRI, 50 mg/kg/day of crocetin isolated from saffron protected heart tissue by inhibiting production of MDA, suppressing expression of TNF‐α, and stopping cells apoptosis (Wang et al., 2014). The findings adapted from the study conducted by Efentakis et al. confirmed improving impacts of 60 mg/kg/day of saffron aqueous extract against MIRI in mice. They propounded the involvement of extracellular signal‐regulated protein kinase1/2(ERK1/2), glycogen synthase kinase‐3‐β (GSK3‐β), and nuclear factor erythroid 2‐related factor 2 (Nrf2) signaling pathways in cardioprotective effects of saffron extract (Efentakis et al., 2017). Table 2 summarizes the effect of saffron and its constituents on cardiovascular system.

**TABLE 2:** The effect of saffron and its constituents on cardiovascular system.

Treatment	Kind of study	Doses	Mechanism (s) effects	References
Saffron	Human	100 mg/day	Amelioration of quality of life and appetite	Ahmadikhatir et al. (2022)
Saffron	Animal	30, 60, and 90 mg/kg	Stabilization of atherosclerotic plaques and modulation of inflammatory reactions	Christodoulou et al. (2018)
Saffron	Human	200 mg	Decrease of blood pressure along with the increase of NO and adiponectin level and reduction of endothelin‐1 concentration	Hooshmand‐Moghadam et al. (2021)
Saffron	Human	200 mg	Amelioration of inflammation and reduction of risk factors causing cardiovascular diseases	Mojtahedi et al. (2022)
Saffron	Animal	10, 20 and 40 mg/kg	Decline of systolic blood pressure, mean arterial blood pressure, and heart rate	Plangar et al. (2019)
Crocin	Animal	50, 100 and 200 mg/kg	Mitigation of systolic blood pressure, mean arterial blood pressure, and heart rate	Shafei, Faramarzi, et al. (2017)
Saffron and crocin	Human	30 mg/day	Increase of SITR1 and AMPK expression, decrease of LOX1, NF‐κB and ox‐LDL production, decrement of MCP‐1 level	Abedimanesh et al. (2020)
Saffron	Animal	200, 400 and 800 mg/kg	Regulation of oxidative status and preservation of hemodynamic functions of cardiovascular system	Sachdeva et al. (2012)
Crocin	Animal	20 mg/kg/day	Improvement of hemodynamic of cardiovascular system and reinforcement of antioxidant defense	Goyal et al. (2010)
Crocin	Animal	100 mg/kg	Mitigation of MDA and enhancement of glutathione, SOD, CAT	Kocaman et al. (2021)
Crocin	Animal	20, 40, and 60 mg/kg	Alleviation of myocardial infarction‐mediated injuries via lessening the concentration of MDA and NO and amplifying the SOD activity	Wang et al. (2018)
Crocin	Animal	25 and 50 mg/kg	Alleviation of subchronic diazinon‐triggered cardiotoxicity by modulation of oxidative stress and inhibition of apoptosis	Razavi et al. (2013)
Saffron	Animal	100 mg/kg	Improvement of lethal ventricular arrhythmias through prolonging the effective refractory period of cardiac cells and preventing the oxidative stress	Joukar et al. (2013)
Crocetin	Animal	50 mg/kg	Protection of heart against MIRI by inhibiting production of MDA, suppressing expression of TNF‐α, and stopping cells apoptosis	Wang et al. (2014)
Saffron	Animal	60 mg/kg	Attenuation of MIRI via affecting ERK1/2, GSK3‐β and Nrf2 signaling pathways	Efentakis et al. (2017)

Abbreviations: AMPK, 5′ adenosine monophosphate‐activated protein kinase; CAT, catalase; ERK1/2, extracellular signal‐regulated protein kinase1/2; GSK3‐β, glycogen synthase kinase‐3‐β; LOX1, lectin‐like oxidized low‐density lipoprotein receptor‐1; MCP‐1, monocyte chemoattractant protein‐1; MDA, malondialdehyde; MIRI, myocardial ischemia–reperfusion injury; NF‐κB, nuclear factor kappa B; NO, nitric oxide; Nrf2, nuclear factor erythroid 2‐related factor 2; ox‐LDL, oxidized low‐density lipoprotein; SITR1, sirtuin; SOD, superoxide dismutase; TNF‐α, tumor necrosis factor alpha.

## 5 EFFECTS ON RESPIRATORY SYSTEM

Chronic obstructive pulmonary disease (COPD) is a kind of progressive respiratory system diseases that is characterized by airflow limitation and destruction of lung tissue (Duffy & Criner, 2019). Multiple factors including age, sex, genetic, infections, and smoking have been listed as the causes of the onset and progression of COPD (De Marco et al., 2011; Thomson, 2022). In addition, transcription factors such as NF‐kB and oxidative stress‐caused airways inflammation play a prominent role in induction of COPD (Wang et al., 2017). Therefore, one of the important therapeutic strategies for COPD is the management of inflammation reactions and oxidative stress. Based on this subject, in a randomized, double blind controlled trial, the therapeutic impact of 30 mg/day of crocin supplementation for 12 weeks on COPD patients was checked. According to the biochemical findings, alleviation of COPD symptoms was associated with decline of blood level of total oxidant status and NF‐kB and increment of total antioxidant capacity (Ghobadi et al., 2022). In a study on diabetic rats, 0.25, 0.5, and 0.75 mg/kg/day of safranal prevent respiratory distress via reducing the MDA and NO level and increasing the GSH concentration and SOD and CAT activity in bronchoalveolar lavage fluid and lung tissue (Samarghandian et al., 2014).

Asthma is a condition in which airways become narrow and hyper‐responsiveness (Chapman & Irvin, 2015). Inflammation of airways has a basic contribution in induction of asthma (Khazdair, Anaeigoudari, et al., 2019). Meanwhile, activation of T helper2 (Th2) results in generation of inflammatory mediators, while Th1 can inhibit Th2 and attenuate inflammation causing asthma (Wu et al., 2016). In experimental studies, ovalbumin is used for sensitization of animals and induction of asthma (Tabaa et al., 2022). In sensitized Guinea‐pigs by ovalbumin, 20, 40, and 80 mg/kg/day of hydro‐ethanolic extract of saffron decreased tracheal responses and the level of IL‐4, nitrite and total NO and enhanced interferon gamma (IFN‐γ) and Th1/Th2 ratio (Byrami et al., 2013). In asthmatic rats by ovalbumin, 50, 100, and 200 mg/kg of hydroalcoholic extract of saffron also improved inflammation and reduced hematological parameters including total white blood cells counts, eosinophil percentage, platelet count, and red blood cell count (Vosooghi et al., 2013). Allergic asthma is a type of asthma that is connected to food and drug allergy, allergic rhinitis, and family history (Hu et al., 2021). This chronic respiratory disease is associated with Th2‐mediated immune responses (León & Ballesteros‐Tato, 2021). In a triple, randomized placebo controlled trial, use of 100 mg/day of saffron ameliorated spirometry test factors such as forced expiratory volume in first second (FEV1), forced vital capacity (FVC), FEV1/FVC ratio, and forced expiratory flow 25%–75% (FEF 25–75) and decreased high‐sensitivity C‐reactive protein (hs‐CRP) and anti‐heat shock protein (anti‐HSP) 70 concentration in patients with allergic asthma (Hosseini et al., 2018). In a mice model of allergic asthma induced by ovalbumin, 25 mg/kg of crocin alleviated the asthma symptoms through down‐regulating the expression of inflammatory cytokines such as IL‐4 and IL‐13 and modulating oxidative stress status (Yosri et al., 2017). Table 3 exhibits the effect of saffron and its constituents on respiratory system.

**TABLE 3:** The effect of saffron and its constituents on respiratory system.

Treatment	Kind of study	Doses	Mechanism (s) effects	References
Crocin	Human	30 mg	Decline of blood level of total oxidant status and NF‐kB and increment of total antioxidant capacity	Ghobadi et al. (2022)
Safranal	Animal	0.25, 0.5, and 0.75 mg/kg	Prevention of respiratory distress via reducing the MDA and NO level and increasing the GSH concentration and SOD and CAT activity	Samarghandian et al. (2014)
Saffron	Animal	20, 40, and 80 mg/kg	Decrease of tracheal responses and the level of IL‐4, nitrite and total NO and enhancement of IFN‐γ and Th1/Th2 ratio	Byrami et al. (2013)
Saffron	Animal	50, 100, and 200 mg/kg	Improvement of inflammation and reduction of hematological parameters including total white blood cells counts, eosinophil percentage, platelet count and red blood cell count	Vosooghi et al. (2013)
Saffron	Human	100 mg	Amelioration of spirometry test factors such as FEV1, FVC, FEV1/FVC ratio and FEF 25–75 and decrease of hs‐CRP and anti‐HSP70 concentration	Hosseini et al. (2018)
Crocin	Animal	25 mg/kg	Alleviation of asthma symptoms through down‐regulating the expression of inflammatory cytokines and modulating oxidative stress status	Yosri et al. (2017)

Abbreviations: CAT, catalase; FEF, forced expiratory flow; FEV1, forced expiratory volume in first second; FVC, forced vital capacity; GSH, glutathione; hs‐CRP, high‐sensitivity C‐reactive protein; HSP, heat shock protein.; IFN‐γ, interferon gamma; IL, interleukin; MDA, malondialdehyde; NF‐kB, nuclear factor kappa B; NO, nitric oxide; SOD, superoxide dismutase; Th, T helper.

## 6 EFFECTS ON GASTROINTESTINAL TRACT

Inflammatory bowel disease (IBD) is a chronic inflammatory disorder of the gastrointestinal (GI) tract which its prevalence is enhancing in worldwide. Crohn's disease (CD) and ulcerative colitis are two clinical subtypes of IBD with the same pathogenesis (Seyedian et al., 2019). It has been found that dysfunction of immune system, genetic and environmental factors, and gut microbiota have an important role in pathogenesis of IBD (Ananthakrishnan, 2015). The clinical and experimental evidence also reveals over‐expression of serotonin or 5‐hydroxytryptamine (5‐HT) by enterochromaffin (EC) cells in IBD (Damen et al., 2013). In addition, it has been recognized that alteration in 5HT content has an eminent role in production of inflammatory mediators (Regmi et al., 2014). Banskota et al. examined pretreatment effect with 10 and 20 mg/kg of saffron in a mice model of colitis induced by dextran sulfate sodium (DSS). They conclude that saffron suppressed 5HT, IL‐1β, IL‐6, and TNF‐α secretion through inhibiting NF‐κB expression. This prophylactic effect of saffron was accompanied with preserving the variety of the gut microbiota and increasing the short‐chain fatty acids (Banskota et al., 2021). In another study on mice, 20 mg/kg of saffron ameliorated colitis excited by DSS by increasing anti‐inflammatory cytokines such as IL‐10 and activating Nrf2 signaling pathway (Singh et al., 2021). Surprisingly, the results of the study of Feng et al. (2022) indicated that 10 and 40 mg/kg of crocetin elicited from saffron overturned intestinal homeostasis and postponed recovery period of DSS‐provoked colitis in mice by exacerbating inflammation and disturbing gut microbiota status.

Gastric cancer is one of the main causes of causing death in different regions of the world. Surgery, radiotherapy, and chemotherapy are methods selected for treatment of gastric cancer (Liang et al., 2020). Besides of these procedures, use of phytochemicals and medicinal plants has also been recommended to cure the gastric cancer (Hassanalilou et al., 2019). Based on this, the effect of intraperitoneal administration of 100, 150, and 175 mg/kg of aqueous extract of saffron on 1‐Methyl‐3‐nitro‐1‐nitrosoguanidine‐caused gastric cancer in rats was evaluated. Pathological results demonstrated that saffron extract dose‐dependently exerted positive therapeutic effects on cancerous rats. Gastro‐protective impacts of saffron are attributed to antioxidant properties (Bathaie et al., 2013). In study of Tamaddonfard et al. (2019), 0.063, 0.25, and 1 mg/kg of safranal protected rats against indomethacin‐prompted gastric ulcer via normalizing oxidative stress status, inhibiting inflammatory reactions and preventing programmed cell death. Table 4 displays the effect of saffron and its constituents on gastrointestinal tract.

**TABLE 4:** The effect of saffron and its constituents on gastrointestinal tract.

Treatment	Kind of study	Doses	Mechanism (s) effects	References
Saffron	Animal	10 and 20 mg/kg	Suppression of 5HT, IL‐1β, IL‐6, and TNF‐α secretion through inhibiting NF‐κB expression. Preservation of the variety of the gut microbiota and increasing the short‐chain fatty acids	Banskota et al. (2021)
Saffron	Animal	20 mg/kg	Increase of anti‐inflammatory cytokines such as IL‐10 and activating Nrf2 signaling pathway	Singh et al. (2021)
Crocetin	Animal	10 and 40 mg/kg	Disturbance of intestinal homeostasis and postponement of recovery period of DSS‐provoked colitis by exacerbating inflammation and disturbing gut microbiota status	Feng et al. (2022)
Saffron	Animal	100, 150, and 175 mg/kg	Exertion of positive therapeutic effects on gastric cancer due to antioxidant properties.	Bathaie et al. (2013)
Safranal	Animal	0.063, 0.25, and 1 mg/kg	Protection of gastric ulcer via normalizing oxidative stress status, inhibiting inflammatory reactions, and preventing programmed cell death	Tamaddonfard et al. (2019)

Abbreviations: 5HT, 5‐hydroxytryptamine; IL, interleukin; NF‐κB, nuclear factor kappa B; Nrf2, nuclear factor erythroid 2‐related factor 2.; TNF‐α, tumor necrosis factor alpha.

## 7 EFFECT ON URINARY SYSTEM

Diabetic nephropathy (DN) is a chronic disturbance of kidney function which happens in individuals with diabetes mellitus (Samsu, 2021). Although pathogenesis of DN is complex and multifactorial, inflammation, and oxidative stress have been demonstrated to have a key role to the induction and progression of DN (Winiarska et al., 2021). The results of numerous of studies emphasize the herbal medicines which have positive effects on diabetes mellitus could alleviate DN symptoms (Liu et al., 2022). It has been reported that, daily oral administration of 20 mg/kg of crocin for 8 weeks improved DN in diabetic rats. Treatment with crocin led to increased level of antioxidant biomarkers such as SOD, CAT, and GSH and decreased concentration of MDA and IL‐6 in kidney tissue of rats (Abou‐Hany et al., 2018a). The protective effect of 50 mg/kg of crocin against nephropathy in mice also was accompanied with the down‐regulation of NF‐kB via the activation of Nrf2 signaling pathway (Qiu et al., 2020). In human research performed by Moravej Aleali et al. (2019), 15 mg of saffron modified blood lipid profile, improved liver and renal function in patients with type 2 diabetes mellitus. Samarghandian et al. also documented the beneficial effect of 10, 20, and 30 mg/kg/day of crocin on renal functional parameters in aged rats' kidney. The reo‐protective effect of crocin was carried out due to the adjustment of oxidative stress condition and suppression of inflammatory cytokines production (Samarghandian et al., 2016). Stress is a condition created by different stimuli that imbalances harmony between oxidant and antioxidant system (Yisireyili et al., 2019). Detrimental impacts of oxidative stress resulting from chronic stress on kidneys have been reported (Xu et al., 2022). In an study on rats, systemic injection of 30 mg/kg of saffron extract and 30 mg/kg of crocin could reduce MDA content and elevate the activity of antioxidant enzymes including glutathione peroxidase (GPx), glutathione reductase (GR) and SOD and untimely protect kidneys, liver and brain against chronic restraint stress‐induced oxidative stress (Bandegi et al., 2014). It has also been indicated that 167.5 and 335 mg/kg/day of saffron petal extract for 8 weeks rescued kidney tissue of rats from injuries caused by ethyl alcohol. Ameliorating effect of saffron was attributed to its anti‐inflammatory and antioxidant properties (Azizi et al., 2019).

Renal ischemia which can be a consequent of hypertension and renal transplantation applies hypoxic oxidative damage and inflammation to kidneys (Greite et al., 2018). Mohamoudzadeh et al. used 5, 10, and 20 mg/kg of hydro‐ethanolic extract of saffron against acute kidney injury induced by ischemia/reperfusion (IR) in rats. Their results exhibited that saffron extract dose‐dependently mitigated the level of creatinine, MDA, TNF‐α, intercellular adhesion molecule‐1, and leukocyte filtration (Mahmoudzadeh et al., 2017). In a rat model of unilateral renal IR, oral administration of 20 mg/kg of crocin for 7 days also lightened renal injuries by attenuating oxidative stress and inflammatory responses. Attention to the results of this study demonstrates that improvement of renal function indexes was associated with the level enhanced of SOD and CAT activity and glutathione (GSH) and decreased concentration of MDA and IL‐6 in rats exposed by corcin (Abou‐Hany et al., 2018b).

Doxorubicin is a cytotoxic antibiotic with anticancer properties. It suppresses the activity of topoisomerase II and disturbs gene transcription resulting in finally to cell death (Shafei, El‐Bakly, et al., 2017). Additionally, it has been documented that doxorubicin induces nephrotoxicity by increasing the production of ROS (Rafiee et al., 2020). In an animal study achieved by Hussain et al. (2021), 100 mg/kg of crocin for 3 weeks quieted doxorubicin‐stimulated nephrotoxicity in rats by exerting antioxidant effect and down‐regulating the production of NF‐κB, TNF‐α, inducible nitric oxide synthase (iNOS), and cyclooxygenase‐2 (COX2). Aminoglycosides including gentamicin has been listed as a category of antibiotics with murderous effects against aerobic bacteria. One of the known side effects of these antibiotics is nephrotoxicity (Meka Kedir et al., 2022). The toxic impacts of aminoglycosides antibiotics on kidney are imagined to be due to the induction of the generation of free radicals (Pakfetrat et al., 2022). Experimental evidence illustrated that 80 mg/kg of aqueous extract of saffron treated gentamicin‐caused nephrotoxicity. Consistent on the results of this study, saffron ameliorated increased serum level of creatinine, blood urea nitrogen (BUN), and MDA in animals treated by extract of saffron (Ajami et al., 2010). Outcome of the study of Ouahhoud et al. also elucidated that 50 mg/kg of hydro‐ethanolic extracts of saffron exerted protective impacts against gentamicin‐caused reno‐toxicity. Reno‐protective effects of saffron were associated with a significant decline in blood concentration of urea, creatinine, uric acid, and albumin as well as considerable reduction in MDA level of kidney tissue (Ouahhoud et al., 2022). Table 5 highlighted the effect of saffron and its constituents on urinary system.

**TABLE 5:** The effect of saffron and its constituents on urinary system.

Treatment	Kind of study	Doses	Mechanism (s) effects	References
Crocin	Animal	20 mg/kg	Increase of level of antioxidant biomarkers such as SOD, CAT, and GSH and decreased concentration of MDA and IL‐6	Abou‐Hany et al. (2018a)
Crocin	Animal	50 mg/kg	Down‐regulation of NF‐kB via the activation of Nrf2 signaling pathway	Qiu et al. (2020)
Saffron	Human	15 mg	Modification of blood lipid profile, improvement of liver and renal function	Moravej Aleali et al. (2019)
Crocin	Animal	10, 20 and 30 mg/kg	Adjustment of oxidative stress condition and suppression of inflammatory cytokines production	Samarghandian et al. (2016)
Saffron and crocin	Animal	30 mg/kg	Reduction of MDA content and elevation of the activity of antioxidant enzymes including glutathione peroxidase (GPx), glutathione reductase (GR), and SOD	Bandegi et al. (2014)
Saffron	Animal	167.5 and 335 mg/kg	Amelioration of inflammation and oxidative stress	Azizi et al. (2019)
Saffron	Animal	5, 10 and 20 mg/kg	Mitigation of the level of creatinine, MDA, TNF‐α, intercellular adhesion molecule‐1, and leukocyte filtration	Mahmoudzadeh et al. (2017)
Crocin	Animal	20 mg/kg	Enhancement of SOD and CAT activity and glutathione (GSH) and decrease of MDA and IL‐6 concentration	Abou‐Hany et al. (2018b)
Crocin	Animal	100 mg/kg	Down‐regulation of NF‐κB, TNF‐α, iNOS, and COX2 expression	Hussain et al. (2021)
Saffron	Animal	80 mg/kg	Amelioration of increased serum level of creatinine, BUN, and MDA	Ajami et al. (2010)
Saffron	Animal	50 mg/kg	Decline of urea, creatinine, uric acid, and albumin concentration as well as considerable reduction of MDA level	Ouahhoud et al. (2022)

Abbreviations: BUN, blood urea nitrogen; CAT, catalase; COX2, cyclooxygenase‐2; GR, glutathione reductase; IL, interleukin; iNOS, inducible nitric oxide synthase; MDA, malondialdehyde; NF‐kB, nuclear factor kappa B; Nrf2, nuclear factor erythroid 2‐related factor 2 GPx, glutathione peroxidase; SOD, superoxide dismutase; TNF‐α, tumor necrosis factor alpha.

## 8 CONCLUSION

**FIGURE 2:**
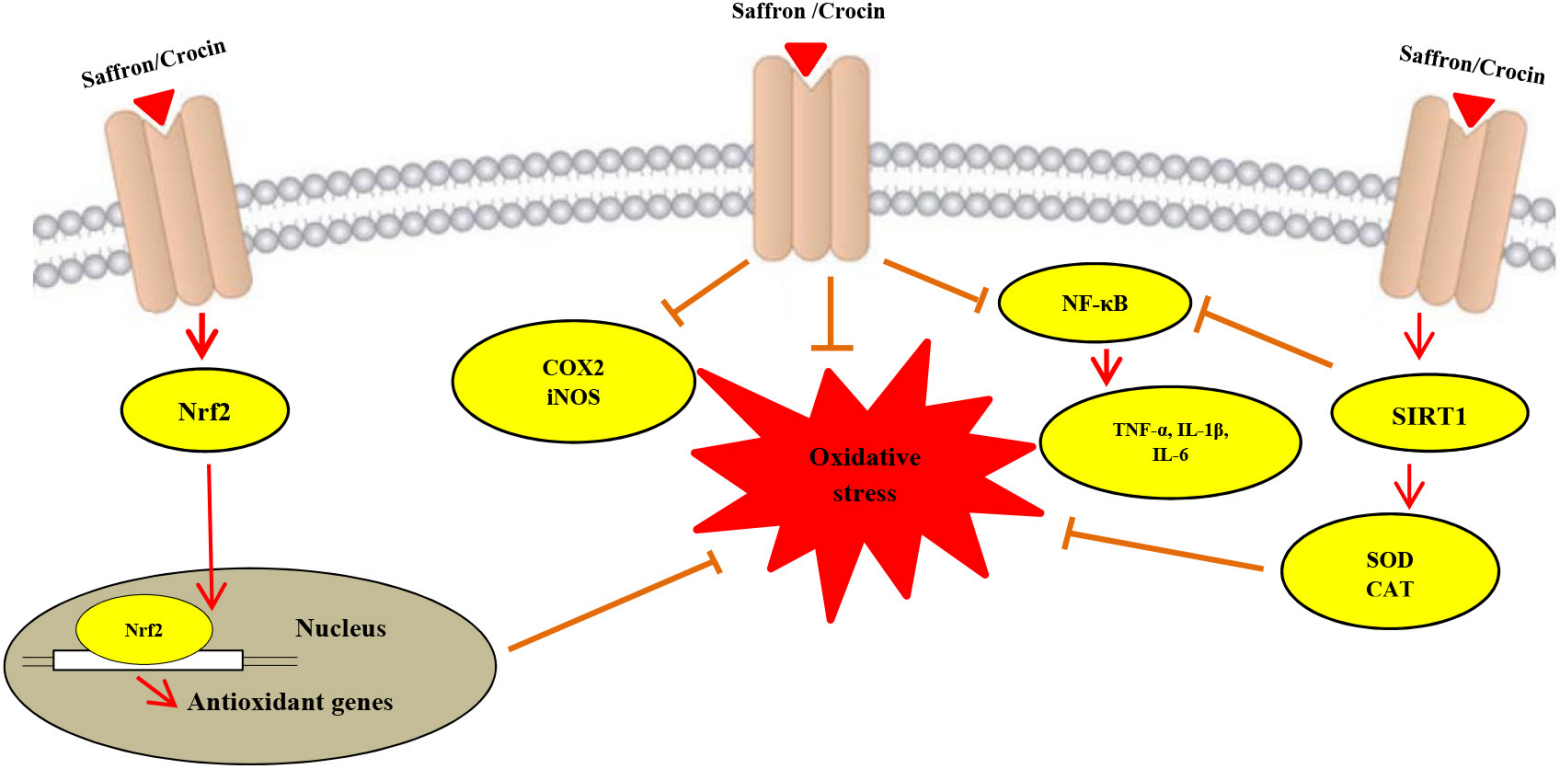
Mechanisms of therapeutic effects of saffron and crocin.

This review quotes the possible therapeutic effects of saffron and its constituents on diverse body systems. The results of the majority of reports demonstrate that the therapeutic impacts of saffron and its constituents have been mediated through inhibiting the generation of oxidative stress indices, amplifying antioxidant capacity, and suppressing the expression of inflammatory mediators. SIRT1 and Nrf2 are two key factors for modulating inflammatory reactions and oxidative stress. Based on outcomes of some studies, a part of protective effects of saffron and crocin derived from it is attributed to the up‐regulation of SIRT1 and Nrf2 signaling. In addition, inhibition of iNOS and COX2 contributes to anti‐inflammatory and antioxidant effects of crocin (Figure 2). However, to elucidate more detailed mechanisms, further studies especially molecular and pathological experiments are required in future.

## AUTHOR CONTRIBUTIONS

Fatemeh Anaeigoudari contributed to study design and collected data; Akbar Anaeigoudari contributed to study design, gathered data and prepared the manuscript; Aliasghar Kheirkhah‐Vakilabad helped to prepare and to edit the manuscript. All authors read the manuscript and approve it.

## FUNDING INFORMATION

No funding information provided.

## CONFLICT OF INTEREST STATEMENT

The authors announce that there is not any conflict of interest.

## ETHICS STATEMENT

None.
